# Calcium Tungstate Microgel Enhances the Delivery and Colonization of Probiotics during Colitis via Intestinal Ecological Niche Occupancy

**DOI:** 10.1021/acscentsci.3c00227

**Published:** 2023-06-21

**Authors:** Jiali Yang, Mengyun Peng, Shaochong Tan, Shengchan Ge, Li Xie, Tonghai Zhou, Wei Liu, Kaixiang Zhang, Zhenzhong Zhang, Junjie Liu, Jinjin Shi

**Affiliations:** † School of Pharmaceutical Sciences, Zhengzhou University, Zhengzhou 450001, P. R. China; ‡ Key Laboratory of Targeting Therapy and Diagnosis for Critical Diseases, Zhengzhou 450001, P. R. China; § Collaborative Innovation Center of New Drug Research and Safety Evaluation, Zhengzhou 450001, P. R. China; ∥ State Key Laboratory of Esophageal Cancer Prevention & Treatment, Zhengzhou, 450001, P. R. China

## Abstract

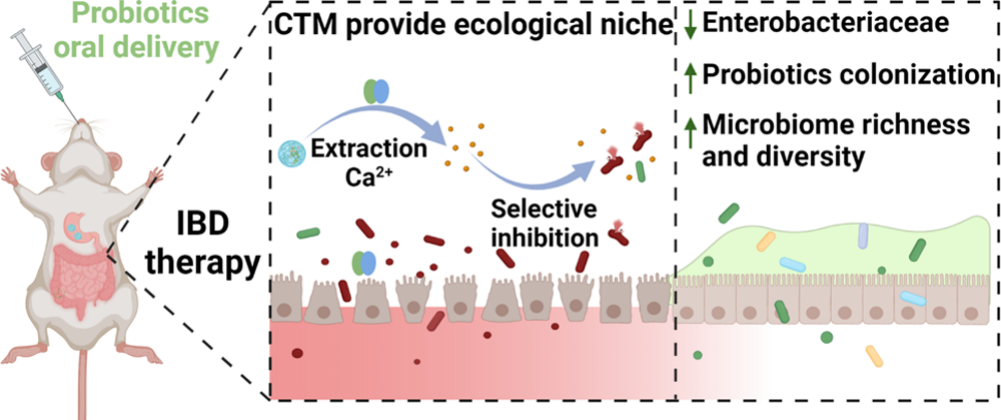

The effective delivery and colonization of probiotics are recommended for therapeutic interventions during colitis, the efficacy of which is hampered by abnormally colonized Enterobacteriaceae at pathological sites. To improve the delivery and colonization of probiotics, a calcium tungstate microgel (CTM)-based oral probiotic delivery system is proposed herein. CTM can selectively disrupt the ecological niche occupied by abnormally expanded Enterobacteriaceae during colitis to facilitate probiotic colonization. In addition, the calcium-binding protein, calprotectin, which is highly expressed in colitis, efficiently extracts calcium from CTM and releases tungsten to inhibit Enterobacteriaceae by displacing molybdenum in the molybdenum enzyme, without affecting the delivered probiotics. Moreover, CTM demonstrated resistance to the harsh environment of the gastrointestinal (GI) tract and to intestinal adhesion. The synergistic reduction of Enterobacteriaceae by 45 times and the increase in probiotic colonization by 25 times, therefore, result in a remarkable treatment for colitis, including restoration of colonic length, effective downregulation of the inflammatory response, restoration of the damaged mucosal barrier, and restoration of gut microbiome homeostasis.

## Abstract 2

Calcium tungstate microgel can selectively disrupt the ecological niche occupied by abnormally expanded Enterobacteriaceae during colitis to facilitate probiotic colonization.

## Introduction

Inflammatory bowel disease (IBD), including Crohn’s disease and ulcerative colitis, is a chronic inflammatory disease of the gastrointestinal (GI) tract.^1,2^ Due to its uncertain multifactorial etiology, the rising incidence of colitis represents a major health burden worldwide.^3^ Immunosuppressive agents, which suppress the immune response and can cause severe side effects due to their nonspecific immunomodulatory effects, are typically the first-line treatment for colitis.^4−7^ As an improvement, the most recent therapies, such as infliximab and adalimumab, have shown high colitis control rates. Still, their large-scale application is severely constrained by drug resistance and high costs.^8,9^ Recently, oral probiotic therapies for the treatment of colitis have received increasing attention. In addition, probiotics have been used clinically to treat antimicrobial-induced colitis and diarrhea.^10^ Previous studies demonstrated the significance of probiotics in maintaining intestinal barrier function and modulating mucosal and systemic immune responses.^11−13^ Therefore, probiotic delivery to the microbiota is a promising preventative and therapeutic approach for colitis.

There are still considerable obstacles to probiotics delivery.^14,15^ The harsh physiological environment of the GI tract, such as gastric acid and bile salts, can severely damage probiotics.^16^ In addition, the continuous peristalsis of the GI tract can accelerate their elimination from the intestine.^17^ Therefore, there is a growing interest in combining drug delivery methods with the microbiome domain to enhance probiotic delivery.^18−22^ Various innovative materials, such as liposomes,^23^ polymer gels,^24−26^ polymer micelles,^27^ bacterial biofilms,^28,29^ and cell membranes,^30^ have been studied to overcome these obstacles during the oral delivery of probiotics. However, these efforts have only focused on protecting probiotics from GI damage under physiological conditions, ignoring the fact that the pathological condition of colitis can also severely impede the colonization of probiotic.^31−33^ Notably, IBD is frequently associated with dysbiosis and is characterized by alterations in the intestinal microbial community, including the expansion of Enterobacteriaceae.^34−38^ In disease states, the massive occupation of ecological niches by Enterobacteriaceae severely restricts the colonization of probiotics. Thus, achieving efficient colonization by disrupting the ecological niche of harmful bacteria presents a formidable challenge.^38^ Therefore, there is an urgent need to develop new strategies to overcome these limitations and improve oral probiotic therapy.

**Scheme 1:**
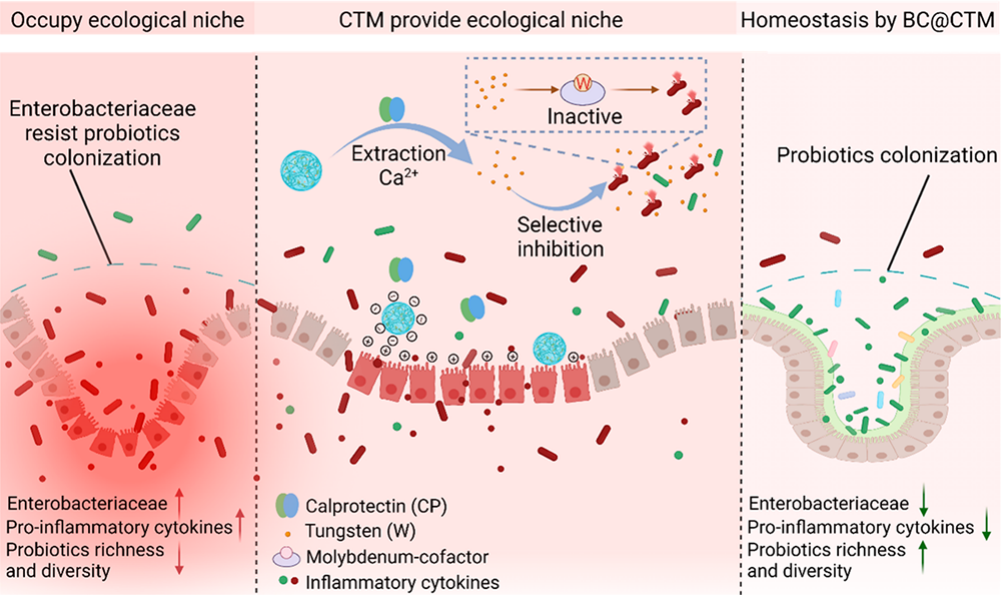
Schematic Diagram of the Mechanism of BC@CTM Treatment for Colitis Oral administration of BC@CTM has a protective effect on the hostile gastrointestinal environment and considerably prolongs the retention time of probiotics in the intestine. CP, which is highly expressed at the colitis site, triggers the release of tungsten by depriving calcium of CTM, thereby inhibiting the growth of Enterobacteriaceae by displacing molybdenum in molybdopterin cofactors, providing additional ecological niches for the delivered probiotics. More probiotic colonization restores the richness and diversity of the intestinal microbiota in colitis, inhibiting inflammation, demonstrating a significant therapeutic effect in colonization.

Herein, we demonstrate calcium tungsten microgels (CTM) to effectively deliver and colonize probiotics in colitis (Scheme 1). During colitis, Enterobacteriaceae proliferate and occupy ecological niches, thereby preventing the colonization of probiotics. Herein, we found that calprotectin (CP), highly expressed at colitis sites,^39,40^ triggers the tungsten release by depriving calcium of CTM, thereby inhibiting the growth of Enterobacteriaceae by displacing molybdenum in the molybdenum pterin cofactor, while having no significant effect on the delivered probiotics. Notably, the selective killing effect disrupts the ecological niche occupied by recalcitrant Enterobacteriaceae in colitis and creates additional ecological niches for probiotics. Thus, in dextran sulfate sodium (DSS)-induced colitis, CTM-encapsulated *Bacillus coagulans* (BC@CTM) effectively inhibited harmful bacteria and promoted probiotic colonization. Additionally, it restored the expression of proteins associated with tight junctions and the function of the intestinal barrier, exerting significant anti-inflammatory effects. The current study, thus, offers a simple strategy to withstand the harsh GI environment and provide additional ecological niches for probiotics colonization during colitis treatment.

## Results and Discussion

### Preparation and Characterization of CTM

**Figure 1:**
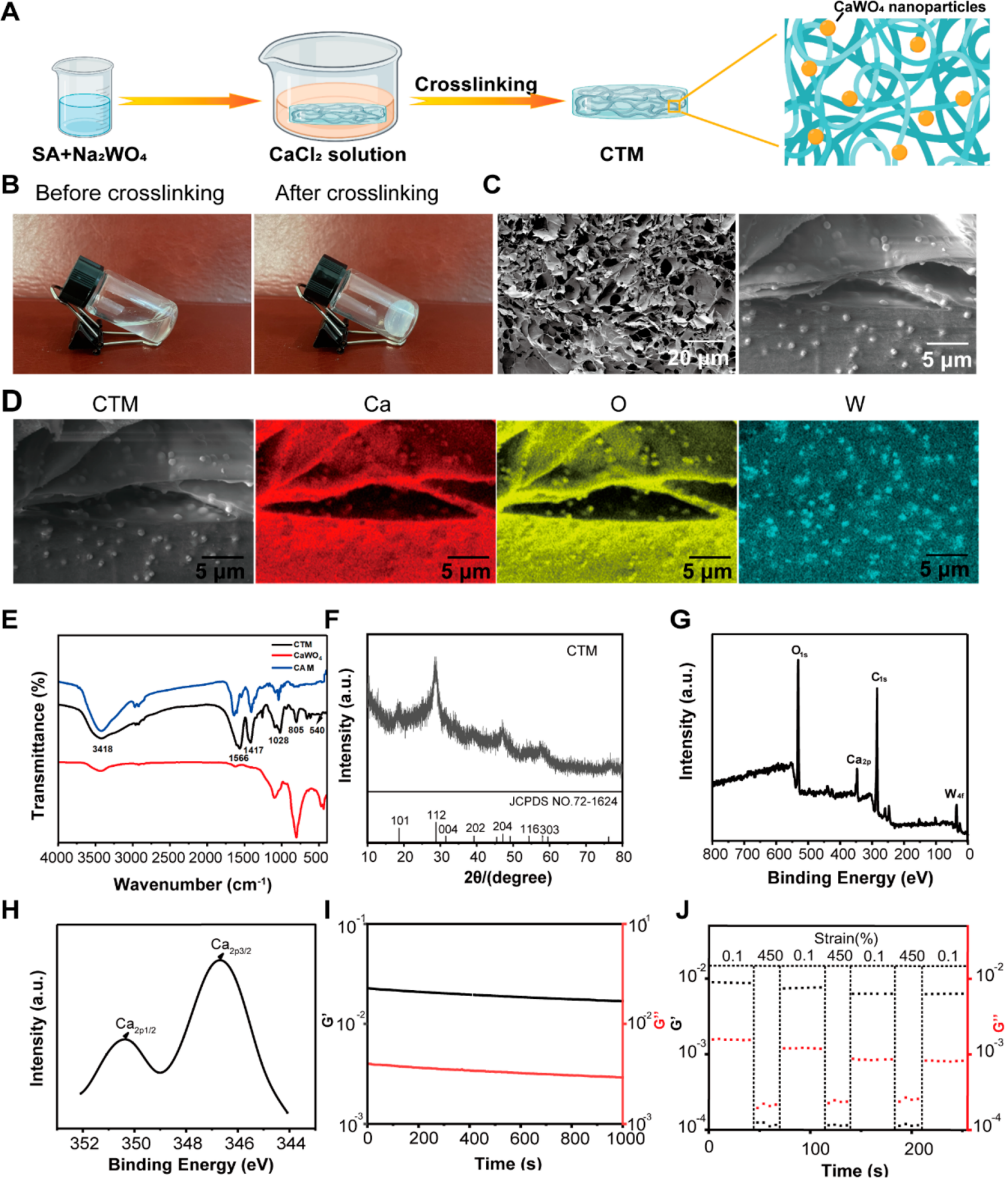
Preparation and characterization of CTM. (A) Schematic illustration of CTM synthesis. The hydrogel was prepared from sodium alginate and tungstate and embedded with CaWO_4_ nanoparticles using a one-step approach. (B) Representative images of CTM before and after gelation. (C) Representative SEM images of CTM. (D) SEM and EDS images of CTM. (E and F) FT-IR and XRD spectra of CTM. (G and H) XPS spectra of CTM: (G) survey spectra, (H) Ca 2p electron level. (I) Time-dependent rheology measurement of the CTM. (J) Thixotropic experiment by continuous step strain measurement of the CTM.

CTM was prepared via a facile and eco-friendly one-step crosslinking process (Figure 1A).^41^ The sodium alginate and sodium tungstate mixture was initially fluid and solidified to CTM (Figure 1B). Scanning electron microscopy (SEM) was used to characterize the surface morphology and microstructure of CTM. CTM has a porous network structure with nondirectional cavities of varying sizes. When compared to calcium alginate microgels (CAM) without calcium tungstate (CaWO_4_) nanoparticles, herein, the particles tended to adhere to the network branches or become embedded in the calcium alginate network beneath. The spherical particles had diameters of approximately 600–700 nm (Figures 1C and S1). The energy dispersive spectrometer (EDS) images showed a distinct surface distribution of O, Ca, and W, indicating that CaWO_4_ nanoparticles were successfully synthesized via the crosslinking reaction (Figure 1D). Figure 1E depicts the Fourier transform-infrared (FT-IR) spectroscopic analysis of CTM compared to CaWO_4_ and CAM in the wavenumber range of 4000–450 cm^–1^. Compared to CaWO_4_ nanoparticles, the CTM’s spectral profile in the range 4000–1000 cm^–1^ was nearly identical to that of CAM, with minor differences due to the addition of a tungstate. These findings suggest that tungstate does not affect the formation of hydrogels.

Figure 1F depicts indexed XRD patterns for CTM to examine the crystal structures of CaWO_4_ in CTM. The XRD pattern exhibits all of the characteristic diffraction peaks and positions described in JCPDS Card No. 72-1624 for the scheelite system. The strongest reflections at 18.58° (101) and 28.78° (112) belonged to the tetragonal scheelite phase confirming a scheelite-type crystal structure in CaWO_4_. X-ray photoelectron spectroscopy (XPS) was also used to examine the surface chemical of CTM. Accordingly, the 0–800 eV measured spectra revealed that the major elements of CTM comprised C, Ca, O, and W (Figure 1G). Additionally, it showed the Ca 2p electron level of CTM, wherein the two typical values of Ca^2+^ oxidation states, Ca_2p_1/2 (350.4 eV) and Ca_2p_3/2 (346.8 eV), were presumably derived from calcium alginate and CaWO_4_ (Figure 1H). Moreover, the zeta (ζ) potential of the CTM was also measured using dynamic light scattering (DLS). The average ζ potential of CTM was found to be −14.1 ± 0.7 mV (Figure S2). Finally, oscillatory tests were conducted. The storage moduli (*G*′) for all treatments were typically greater than the loss moduli (*G*″), indicating that cross-linking induced a typical solid-like gel behavior (Figures 1I and S3). As evident from the thixotropic behavior of CTM, the hydrogel collapsed at 300% strain (Figure S4). After complete rupture at 450% strain, when the hydrogel was subjected to continuous step strain measurement, both gels recovered to their original state at a constant 0.1% strain (Figure 1J). This demonstrates that CTM has an excellent thixotropic property. All of the above-mentioned results, thus, provide ample evidence for the successful preparation of CTM.

### CTM Specifically Releases Tungsten in the Presence of CP to Inhibit Enterobacteriaceae

**Figure 2:**
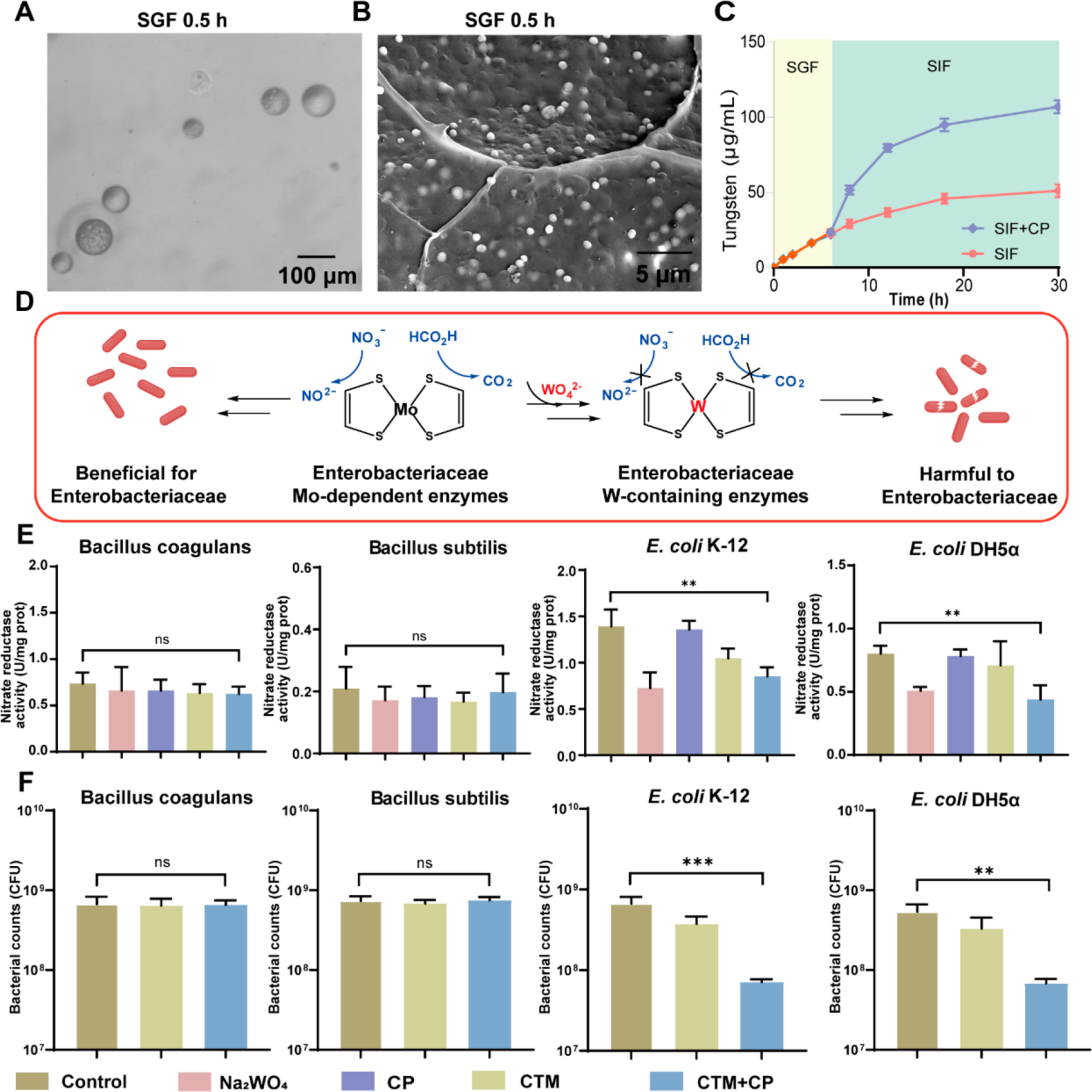
CTM specifically releases tungsten in the presence of CP to inhibit Enterobacteriaceae. (A) Representative confocal laser scanning microscopy images of CTM after 0.5 h of incubation in SGF. (B) Stability of the tungstate nanoparticles after CTM was treated with SGF. (C) Tungsten release from CTM after incubation in SGF for 6 h and continued SIF for 24 h (with or without 50 μg mL^–1^ CP) using ICP-MS. (D) Rational manipulation of gut microbiota by altering molybdenum enzymes. (E) Nitrate reductase activities of BC, BS, *E. coli* K-12, and *E. coli* DH5α were measured in a medium supplemented with sodium nitrate (*n* = 3). (F) The growth of BC, BS, *E. coli* K-12, and *E. coli* DH5α in different treatments under anaerobic conditions (*n* = 3). Data are presented as the means ± SD (*n* = 3). *P < 0.05, **P < 0.01, ***P < 0.001 determined by Student’s *t*-test.

To suit oral delivery, we prepared a spherical CTM via re-emulsification. The CTM had a diameter of approximately 100 μm and contained 8.236 μg mg^–1^ of tungsten (Figure S5). Subsequently, we examined the properties of CTM *in vitro*. To examine the equilibrium swelling behavior of hydrogels at different pH values, samples were swollen at room temperature in buffer solutions at pH 1.2, 6.8, and 7.4. The swelling rate of CTM was observed to increase significantly with increasing pH, reaching a maximum of 1943% at pH = 7.4 (Figure S6). The morphological changes of CTM in simulated gastric and intestinal fluids are shown in Figure S7. After 0.5 h of incubation in simulated gastric fluid (SGF, pH 1.2), CTM retained its intact and compact structure (Figure 2A), and the CaWO_4_ nanoparticles embedded within CTM were also preserved (Figure 2B). This indicates that CTM has a protective effect on CaWO_4_ nanoparticles. After 1 h of incubation in simulated intestinal fluid (SIF), the CaWO_4_ nanoparticles decreased as the CTM expanded and dissolved (Figures S8 and S9). During a 1 h incubation in SIF + CP (50 μg mL^–1^), the CTM mostly dissolved, and the embedded CaWO_4_ nanoparticles disappeared (Figure S10). Thus, we hypothesized that CP could bind calcium from CaWO_4_ in the CTM to release sufficient tungsten when it is highly expressed in colitis. As a result, tungsten release from CTM was detected using inductively coupled-plasma mass spectrometry (ICP-MS) following continuous treatment with SGF for 6 h and SIF for 24 h *in vitro*. Accordingly, CTM released tungsten slowly when treated with SGF and SIF but more efficiently when treated with CP (50 μg mL^–1^) (Figure 2C). We also measured the CP content in the colonic tissue of healthy and colitis mice using ELISA, which were 35.73 and 375.98 μg mL^–1^, respectively (Figure S11). Meanwhile, the previous studies have reported CP at the colitis site exceeded 500 μg mL^–1^, providing a solid foundation for CTM to release adequate tungsten in response to CP *in vivo*.^42,43^ To further simulate the release of tungsten in an inflammatory condition, we selected the concentration of CP as 500 μg mL^–1^. The experimental results showed that the release of tungsten was increased by nearly 13 times when the CP concentration was 500 μg mL^–1^ (Figure S12). Then, we investigated the tungsten concentration in the healthy GI tracts and colitis position. As shown in Figure S13, the tungsten ion content in the healthy and colitis intestines was 13 and 40 μg g^–1^, respectively. These results provide a solid basis for CTM release of sufficient tungsten in response to CP *in vivo*.

Tungsten inhibits the growth of Enterobacteriaceae associated with colitis by inhibiting Enterobacteriaceae-dependent molybdenum enzyme activity (Figure 2D).^44^ To determine if CP-mediated tungsten release could inhibit bacterial nitrate reductase activity, we compared the effects of free tungsten, CP, CTM, and CTM + CP (50 μg mL^–1^). Compared to CTM, CTM + CP significantly inhibited nitrate reductase activity in *E. coli* (K-12 and DH5α), comparable to free tungsten. Conversely, neither CTM nor CTM + CP had any effect on *Bacillus coagulans* (BC) or *Bacillus subtilis* (BS) (Figure 2E). This is because tungsten can replace molybdenum in molybdenum enzymes and inhibit the activity of Enterobacteriaceae-dependent molybdenum enzymes,^34,45^ whereas the growth of probiotics is not dependent on molybdenum enzyme, which means that CTM has a selective inhibitory effect (Figure 2E). Notably, CP had no significant effect on nitrate reductase, suggesting that tungsten primarily inhibits nitrate reductase activity. Furthermore, we examined the anaerobic growth of wild-type *E. coli* strains (K-12 and DH5α) in mucin broth supplemented with sodium nitrate to determine whether the addition of CTM and CP could counteract the fitness advantage of anaerobic respiration *in vitro*. Although *E. coli* grew more rapidly in the presence of electron receptors, such as nitrates, this fitness advantage disappeared when CTM + CP was added (Figure 2F). Therefore, these findings confirm that CTM can release tungsten in the presence of CP in colitis, inhibiting the growth of Enterobacteriaceae while having no significant effect on probiotics, thereby destroying the ecological niche occupied by harmful bacteria *in vivo*.

### Probiotics Encapsulation and the Protection of CTM

**Figure 3:**
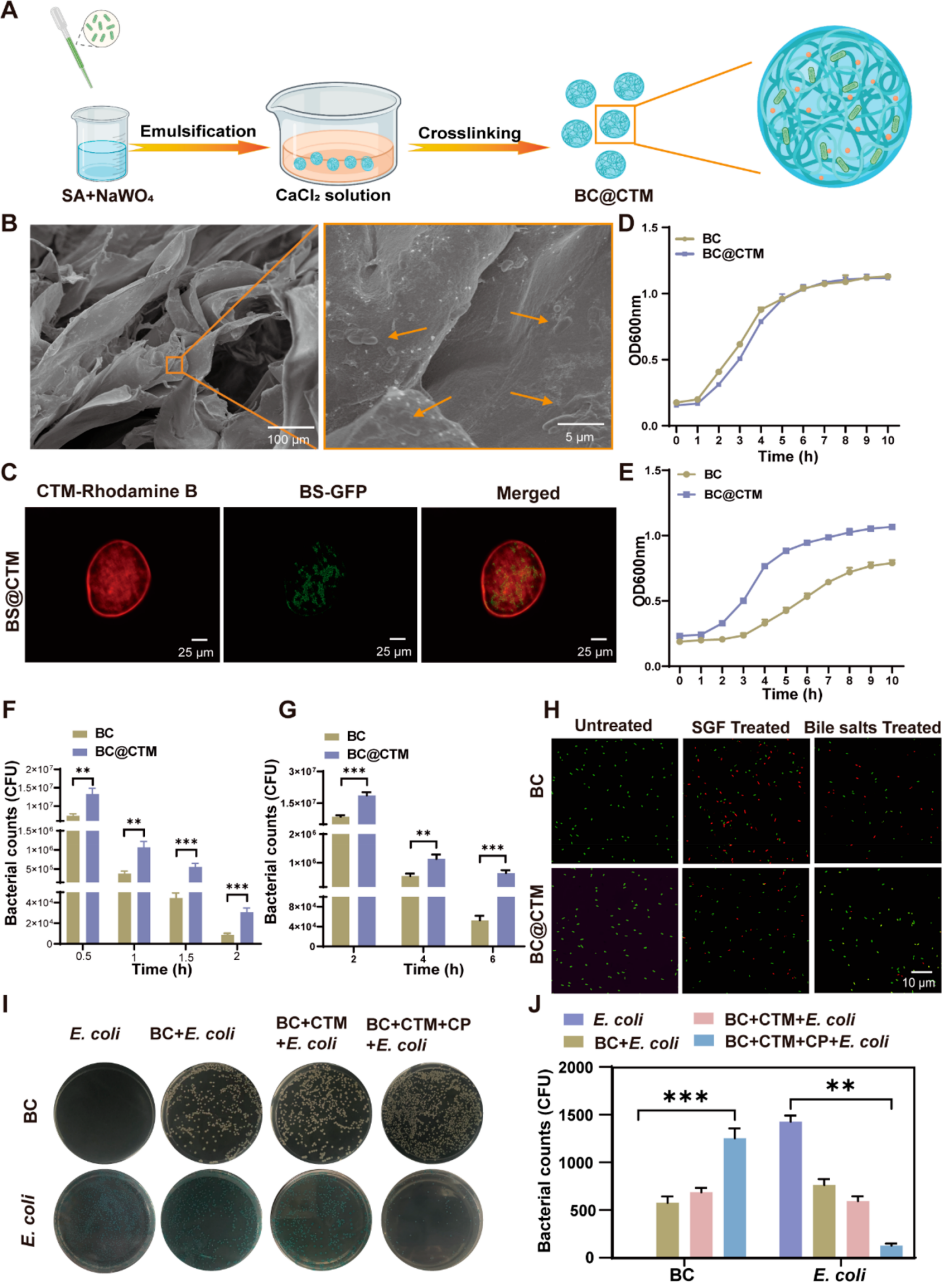
Encapsulation of probiotics and protection of CTM. (A) CTM was loaded with BC via CaCl_2_ solution crosslinking using a w/o emulsion method. (B) SEM images showing the distribution of the probiotics BC in the CTM. (C) The autofluorescent modified probiotic BS-GFP was encapsulated in CTM. (D) Growth curves of BC and BC@CTM in NB medium at 37 °C were recorded every 1 h with a microplate spectrophotometer (*n* = 3). (E) Growth curves of BC and BC@CTM in NB medium after simulated gastric acid and bile salt treatment (*n* = 3). (F–G) Equal amounts of BC and BC@CTM were exposed at 37 °C to (F) SGF and (G) bile salts (0.3 mg mL^–1^) (*n* = 3). (H) The live/dead cell viability assay of BC and BC@CTM before and after treatment in SGF for 0.5 h and bile salts for 1 h. Green for live cells and red for dead cells. (I–J) Number of bacteria in different groups after 24 h of culture in competition with harmful bacteria (*n* = 3). Data are presented as the means ± SD (*n* = 3). *P < 0.05, **P < 0.01, ***P < 0.001 determined by Student’s *t*-test.

Spherical CTMs encapsulating BCs (BC@CTM) were prepared via the re-emulsification with an 87% encapsulation rate (Figure 3A). The encapsulated probiotics were visible in the SEM image (Figure 3B). In addition, confocal laser scanning microscopy (CLSM) confirmed the encapsulation of probiotics within the CTM (Figure 3C). The growth rate of BC in the BC@CTM group was comparable to BC grown in nutrient broth (NB) medium without encapsulation at 37 °C, and the dilution plate counts obtained the same conclusion, indicating that CTM does not affect the growth of BC (Figures 3D and S14). Moreover, the Cell Counting Kit-8 assay (CCK-8 assay) also confirmed that CTM encapsulation does not affect the activity of BC (Figure S15).

Oral probiotics are typically exposed to a harsh GI environment, so we subsequently examined the protective effect of CTM on BC. We first investigated the viability of probiotics after exposure to GI components. With increasing time, BC@CTM grew faster than BC (Figure 3E), indicating that CTM can protect BC from the permeation of gastric acid and bile salts, thereby preventing BC from being killed. In addition, bacterial plate counts indicated that CTM could protect BC from gastric acid and bile salts. After 2 h of incubation in SGF, a large number of viable cells could still be seen in the BC@CTM group. During this period, almost total mortality was observed in the uncoated BC group (Figure 3F). As depicted in Figure 3G shown, the survival rate of the BC group decreased after 2 h of incubation in bile salts (0.3 mg mL^–1^). Notably, even after the incubation time was extended to 6 h, the viable bacteria in the BC@CTM group remained nearly 12 times higher (10^5^ CFU mL^–1^) than that in the BC group. To observe the protective effect of the hydrogel on probiotics, bacteria with different treatments were stained with a live/dead cell viability assay. Many red dead cells were observed in the BC group, whereas most BC@CTM cells were still alive, as shown in Figure 3H.

The harsh physiological environment of the GI tract and, more importantly, the abnormal proliferation of *E. coli* during colitis limit probiotic colonization. Therefore, we investigated whether CTM-encapsulated probiotics could disrupt the ecological niche occupied by harmful bacteria to enhance probiotic colonization. *In vitro* assessment of competitive bacterial colonization (Figure 3I, J) showed that colonization of BC alone was challenging, and BC + CTM colonization was not highly competitive. In contrast, BC + CTM + CP significantly enhanced BC colonization. This indicates that high levels of CP expression in conjunction with CTM release tungsten in colitis, disrupting ecological niches occupied by harmful bacteria and promoting BC colonization. Therefore, all of the aforementioned results demonstrate that CTM could withstand harsh conditions, bind to the highly expressed CP in colitis, promote probiotic colonization by inhibiting the proliferation of harmful bacteria, and facilitate subsequent probiotic colonization *in vivo*.

### Enhanced Delivery and Colonization of BC@CTM in the Mouse Intestine

**Figure 4:**
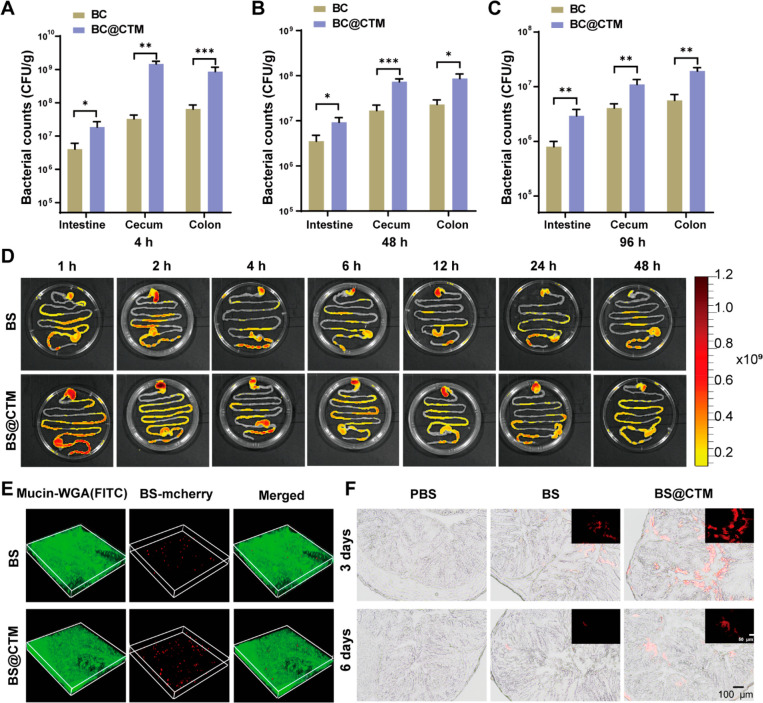
Enhanced delivery and colonization of BC@CTM in the mouse intestine. (A–C) Bacterial counts in various parts of the intestine at (A) 4 h, (B) 48 h, and (C) 96 h after oral administration, respectively. Each mouse was orally dosed with 1 × 10^8^ CFU of BC or BC@CTM and then euthanized at 4, 48, and 96 h. The contents extracted from the tissues were ground and serially diluted with phosphate-buffered saline (PBS), and 40 μL was taken and spread on NB agar medium. The colonies were then incubated overnight at 37°C and counted. (D) Distribution of BS-mCherry and BS-mCherry@CTM in the GI tract within 48 h after oral administration by intestinal transit assay. (E) 3D images of free BS-mCherry, BS-mCherry@CTM in the intestinal mucus attachment of mice. Red: BS-mCherry. Green: mucus stained with FITC-WGA. (F) Long-term intestinal adhesion of free BS-mCherry (red), BS-mCherry@CTM *in vivo*. Typical images of colonic sections on days 3 and 6 after one gavage were obtained by CLSM. Scale bar, 100 μm. Data are presented as the means ± SD (*n* = 3). *P < 0.05, **P < 0.01, ***P < 0.001 determined by Student’s *t*-test.

We also confirmed whether CTM could enhance probiotic colonization in mice. The number of probiotics adhering to the GI tract was quantified via plate counts at different time points after oral administration of 1 × 10^8^ CFU BC@CTM or BC. It was evident that the counts of BC@CTM in the intestinal tract greatly outnumbered those of BC, and that the survival rate of BC@CTM in the GI tract of mice was nearly 14 times that of BC (Figure S16). Figure 4A demonstrates that BC@CTM in the colon was approximately 13-fold higher than BC at 4 h after oral administration. The results were comparable at 48 and 96 h, with an approximately 4-fold and 3.5-fold increase over the BC group (Figure 4B,C). In addition, the oral survival rate was determined by quantifying the total amount of BC colonized in the GI tract. At 4, 48, and 96 h after gavage, the total probiotic amount in the GI tract was 28, 5, and 3-fold higher in the BC@CTM group compared to the BC group, respectively, demonstrating the enhanced survival of probiotics after CTM encapsulation (Figure S17). In addition, gram staining demonstrated that BC@CTM could concentrate bacteria in the intestinal mucosa (Figure S18). To visualize the distribution of BC@CTM or BC in the GI tract, an intestinal transit assay was performed *in vivo* using an animal imaging system. As shown in Figures 4D and S19, CTM encapsulated BS-mCherry expressing red fluorescence (BS-mCherry @CTM) was used for quantification, and the fluorescence intensity of BS-mCherry @CTM in the intestine of mice was higher than that of the BS-mCherry group, which indicated enhanced retention and adhesion of BS-mCherry @CTM in the intestine. In addition, the fluorescence signal of other major organs (heart, liver, spleen, lung, and kidney) was negligible throughout the assay period, indicating that BS-mCherry @CTM was metabolized in the GI tract following gastric administration (Figure S20). Figure 4E depicts increased adhesion of BS-mCherry @CTM to the GI tract as determined by *in vitro* mucosal adhesion assays in intestinal tissues. For long-term observation of intestinal adhesion of BS-mCherry @CTM or BS-mCherry, probiotics in the intestine was observed on day 3 and 6 after one gavage using BS-mCherry. Since no fluorescent signal was observed on day 6, BS had been eliminated from the intestine. Surprisingly, the fluorescence signal of the BS-mCherry @CTM treated group was clearly visible on day 3 and day 6, indicating the long-term adhesion and colonization of BS-mCherry @CTM in the intestine (Figure 4F). The results discussed above indicate that CTM effectively promoted the growth and colonization of probiotics in the intestine. Additionally, it should be mentioned that no side effects were found with oral administration of BC@CTM. The body weight of mice did not differ significantly between the two groups (Figure S21). On day 5 after oral administration, Hematoxylin and Eosin (H&E) staining revealed no histological damage or morphological differences in the intestinal tissues compared to the control group (Figure S22).

### BC@CTM Exerts a Powerful Therapeutic Effect in DSS-Induced Colitis

**Figure 5:**
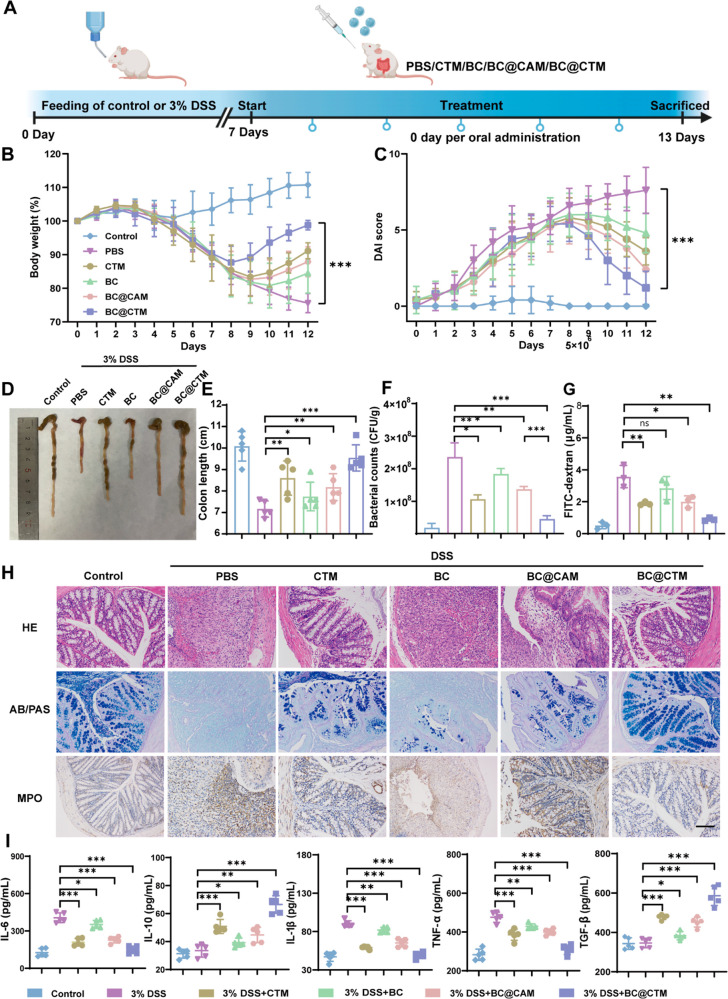
BC@CTM exerts a powerful therapeutic effect in DSS-induced colitis. (A) Procedural diagram of modeling and treatment. Mice were fed with water or water containing 3% DSS for 7 days and then daily treated with CTM, BC, BC@CAM, BC@CTM, or PBS for 5 days. On day 13, these mice were sacrificed. (B) Change in body weight of mice in each group during 13 days (*n* = 5). (C) DAI scores of mice in different groups (*n* = 5). (D) Representative colonic tissue images of mice in each group. (E) Length of the colon in each group of mice (*n* = 5). (F) On day 13, feces were collected and cultured overnight with *E. coli* selective agar medium for *E. coli* colony counting (*n* = 5). (G) On day 13, the intestinal barrier function was assessed *in vivo* in each group of mice by oral administration of 4 kDa FITC-dextran, and fluorescence intensity in serum was measured 4 h later. (H) Representative images of HE, AB/APS, and MPO staining in the colon of mice in each group. Scale bar, 100 μm. (I) On day 13, pro and anti-inflammatory cytokines levels in colonic tissue were analyzed (*n* = 5). Data are presented as mean ± SEM, *P < 0.05, **P < 0.01, ***P < 0.001 determined by Student’s *t*-test.

DSS-induced colitis is one of the most frequently used animal models for simulating IBD. We next evaluated the efficacy of BC@CTM in treating DSS-induced colitis in mice. Figure 5A depicts the experimental design for studying colitis in mice. The DSS-treated mice experienced a significant weight loss compared to the control group. The body weight of BC@CTM-treated mice was quickly regained, while the calcium alginate microgel encapsulated BC without CaWO_4_ nanoparticles (BC@CAM), BC, and PBS-treated mice failed to prevent weight loss (Figure 5B). As shown in Figure 5C, the disease activity index (DAI) quantified the severity of colitis by assigning scores for weight loss, fecal consistency, and fecal bleeding. The DAI values in the control group tended to stabilize, whereas those in the DSS-treated increased rapidly, indicating a more severe form of colitis. On day 12, compared to the DSS-treated group (7.6 ± 0.24), the DAI values of the CTM, BC, BC@CAM, and BC@CTM-treated groups were reduced by 68.46%, 36.84%, 52.63%, and 84.21%, respectively, indicating that BC@CTM was the most effective in alleviating the severity of colitis. Length and condition of the colon are visual indicators of the severity of DSS-induced colitis. Residual blood stool was evident in the colon photographs of the BC group, while the situation was significantly improved after BC@CTM treatment. Moreover, the colon length in the BC@CTM-treated group (9.54 ± 0.21 cm) was significantly longer compared to the BC@CAM-treated group (8.18 ± 0.34 cm) and the BC group (7.48 ± 0.17 cm), respectively, indicating that BC@CTM protected colonic tissue from DSS-induced injury (Figure 5D,E). Given that the expansion of Enterobacteriaceae populations is directly associated with colitis, the abundance of *E. coli* in feces was quantified, as shown in Figure 5F. The abundance of *E. coli* was dramatically reduced in mice treated with BC@CTM compared to BC@CAM (*P* < 0.001). This also indicates that CTM containing CaWO_4_ inhibits Enterobacteriaceae significantly and provides abundant ecological niches for the probiotics delivered. Moreover, when compared to the PBS, BC, and BC@CAM-treated groups, the BC@CTM-treated group effectively prevented systemic exposure of fluorescein isothiocyanate (FITC)-dextran after oral administration in mice with DSS-induced colitis (*P* < 0.01, Figure 5G), indicating the restoration of intestinal barrier function. Additionally, we performed H&E staining experiments to evaluate the histopathological changes. H&E-stained colon sections showed severe histological damage with irregular epithelial structures, loss of crypt foci, and depletion of small intestinal cells in the DSS-treated group (Figure 5H). In contrast, the BC@CTM-treated group exhibited normal colonic histology with intact surface epithelium and crypt structures. These findings corresponded with histopathological scores, where the DSS-treated group had the highest scores, and the BC@CTM intervention significantly decreased them (Figure S23). The immunofluorescence staining images demonstrated that administration of BC@CTM to DSS-induced colitis mice normalized the expression pattern of occludin-1 and ZO-1, two tightly linked related proteins that play a crucial role in intestinal homeostasis (Figure S24). Similar to the control group, alcian blue/periodic acid Schiff staining images demonstrated that BC@CTM treatment significantly repaired the mucus layer (Figure 5H). In addition, myeloperoxidase (MPO), a peroxidase associated with the presence of neutrophils in the mucosa and submucosa of inflammatory tissues, was significantly reduced after BC@CTM treatment (Figure 5H). In contrast, reactive oxygen species (ROS) levels were significantly lower in the BC@CTM-treated group than in the DSS, CTM, BC, and BC@CAM-treated groups (Figure S24). The reduced levels of MPO and ROS indicate that BC@CTM could effectively reduce intestinal inflammation. In addition, the expression of pro-inflammatory cytokines IL-6, IL-1β, and TNF-α was reduced in the BC@CTM-treated group, whereas the expression of anti-inflammatory cytokines TGF-β and IL-10 increased significantly, indicating that BC@CTM ameliorated inflammation (Figure 5I). In conclusion, these results indicate that BC@CTM has a significantly greater therapeutic effect on DSS-induced colitis in mice than BC, CTM, or BC@CAM.

### Modulation of the Gut Microbiome

**Figure 6:**
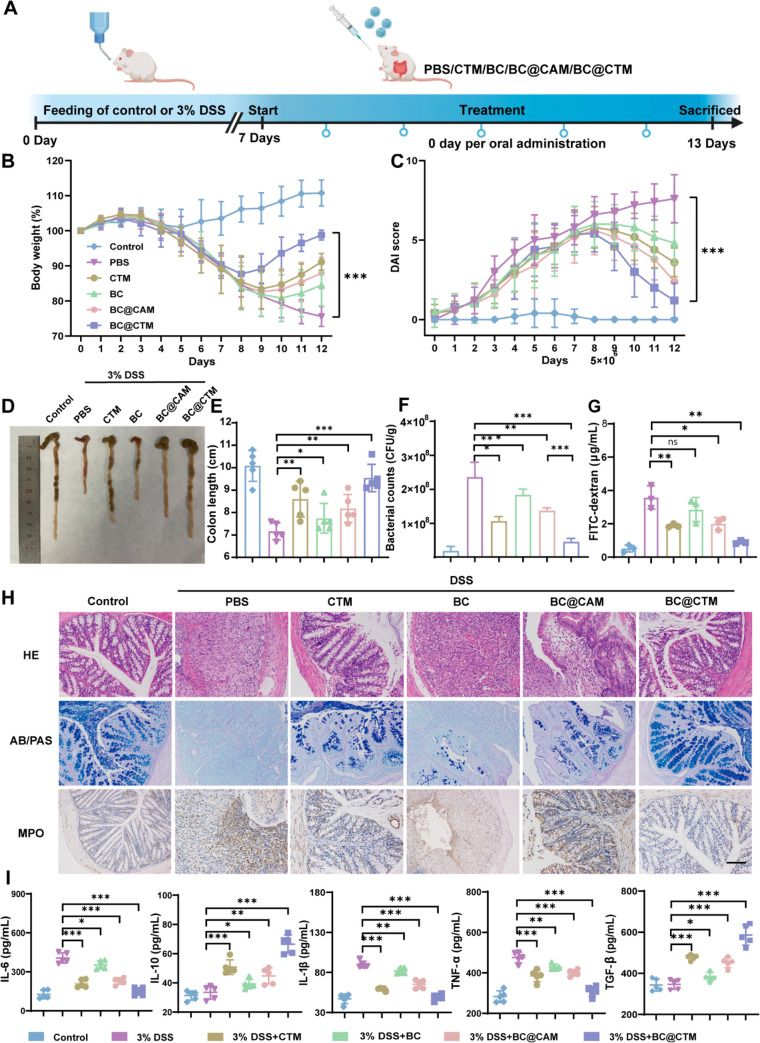
Modulation of the gut microbiome. BALB/c mice were fed water containing 3% DSS for 7 days, and the mice were treated as shown in Figure 5. Following treatment, the feces collected from each group of mice were analyzed for the gut microbiome via 16S rRNA sequencing. (A) UPGMA clustering tree analysis. (B) Relative abundance of the gut microbiome at the order level. (C) Effect of BC@CTM, PBS, and DSS on gut microbiota at the genus level using the ternary graph method. (D–E) Analysis of species with significant differences using the *t*-test.

Dysbiosis of the gut microbiota is closely associated with colitis, including the abnormal proliferation of Enterobacteriaceae and loss of beneficial bacterial taxa. Therefore, we investigated whether BC@CTM treatment could enhance probiotics colonization by disrupting the ecological niche occupied by harmful bacteria. Accordingly, we performed 16S rRNA gene sequencing on fecal samples from mice following treatments. The nonmetric multidimensional scaling (NMDS) plots showed that DSS-colitis mice treated with BC@CTM had a distinct gut microbiota profile compared to other treatment groups (Figure 6A). UPGMA clustering analysis was performed using the unweighted UniFrac distance matrix, and the clustering results were combined with the relative abundance of species at the Phylum level for each sample. The clustering analysis revealed that the gut microbiome of BC@CTM-treated DSS-colitis mice was more similar to that of control groups (Figure 6A). Similar results were obtained when the microbial composition was analyzed at the order level, indicating that BC@CTM treatment restored the gut microbiome homeostasis (Figure 6B). Further analysis of the ternary plot and the *t*-test showed that BC@CTM treatment significantly decreased the proportion of harmful bacteria such as Enterobacteriaceae (known to be positively associated with the development of enteritis), while increasing the proportion of the Lachnospiraceae_NK4A136_group (known to play a beneficial role in the production of short-chain fatty acids in animal models of IBD) and Muribaculaceae (known to have a beneficial role in the production of short-chain fatty acids) (Figures 6C–E and S25). LEfSe (LDA effect size) is an analytical tool, typically used for identifying and interpreting high-dimensional biomarkers, thereby facilitating the identification of statistically different biomarkers between groups. The analysis confirmed that the BC@CTM treatment decreased the abundance of harmful bacteria and increased the proportion of probiotics (Figure S26). In addition, it was further confirmed that BC@CTM exerted a better efficacy due to the fact that tungsten broke the ecological niche occupied by harmful bacteria and promoted better colonization of probiotics. In addition, we used tungsten-free hydrogel (CAM) as a control to examine the changes in gut microbiota after treatment. The results are shown in Figures 6E and S27. Compared with BC@CAM, BC@CTM had higher probiotics enrichment and a lower abundance of pathogenic bacteria. These results confirm that the synergistic effect of BC@CTM, increasing oral delivery of probiotics and disrupting the ecological niche occupied by harmful bacteria during colitis, can promote the recovery of healthy intestinal microbiota and exert an excellent therapeutic effect on colitis.

## Discussion and Conclusions

We developed a probiotic delivery system that enhances the delivery and colonization of probiotics in colitis by occupying ecological niches in the intestine (Scheme 1). IBD is largely caused by an imbalance in the gut-associated microbial community, characterized by an increase in Enterobacteriaceae and a decrease in probiotics.^35,46,47^ Increasing evidence suggests that probiotics delivery can restore gut microbiota homeostasis to treat IBD.^48−54^ However, in colitis, parthenogenic anaerobic Enterobacteriaceae proliferate and occupy ecological niches, restricting the colonization of probiotics.^34−36^ Previous studies have attempted to administer antibiotics first, followed by probiotics, to create ecological niches for probiotic colonization.^55^ However, the use of antibiotics in IBD treatment also removes the beneficial intestinal microbes;^56^ this nonspecific strategy thus increases the risk expansion of harmful bacteria and antibiotic resistance. Furthermore, the increase in antibiotic-resistant bacteria has emerged as one of the greatest threats to human health and a serious economic and societal burden for both developed and developing countries.^34,38,45^ Thus, targeted inhibition of the growth of harmful bacteria and regulating the balance of the gut microbiota present interesting strategies to intervene in the progression of IBD.^34,44^ This study demonstrates that the growth of Enterobacteriaceae can be restricted precisely and effectively with oral CTM without affecting the growth of probiotics, thereby providing ecological niches for probiotic colonization and enhancing therapeutic efficacy. We used CTM as a probiotics carrier to efficiently load and deliver probiotics, protecting the loaded probiotics from destruction by gastric acid to increase their bioavailability. In addition, the negative surface charge of the CTM carrier can bind to positively charged mucin at the site of colonic disease to increase the retention time of probiotics in the intestinal tract. CP could bind to calcium in CTM, triggering CTM disassembly to release tungsten (increased release by 3 times). It is possible that CP is activated by binding to calcium in CTM to exert a host immunomodulatory effect.^57^ It is the specific release of tungsten ions from the inflammation site reduces the nonspecific distribution of tungsten ions that improve the targeting and utilization of the drug, as well as reduces the toxic effects of tungsten ions. BC@CTM has been shown to be effective against DSS-induced colitis. Additionally, it exhibited strong anti-inflammatory effects, upregulated the expression levels of tight junction-related proteins in the colon, restored intestinal barrier function, and protected the epithelium from apoptosis, suggesting its excellent therapeutic effect on intestinal inflammatory diseases. Moreover, both *in vitro* and *in vivo* studies of BC@CTM treatment revealed no adverse effects. BC@CTM also altered the intestinal microbiome, reducing the relative abundance of Enterobacteriaceae (known to be positively associated with the development of enteritis), while increasing the relative abundance of Lachnospiraceae_NK4A136_group (known to produce short-chain fatty acids in animal models of IBD) and Muribaculaceae (known to play a beneficial role in IBD). Our study is limited to a mouse model of DSS-induced acute colitis and requires validation in other advanced preclinical models of IBD. These results suggest that BC@CTM effectively inhibits Enterobacteriaceae involved in colitis, provides ecological niches for probiotics, and protects the colonic epithelium from inflammation while restoring the disrupted intestinal barrier function and microbiome. Thus, the CTM reported here could be a promising probiotics delivery strategy to improve probiotic oral delivery and probiotics colonization efficiency by disrupting the ecological niche occupied by harmful bacteria. Accordingly, this study is highly likely to provide a new perspective on the intestinal colonization of probiotics in treating various diseases.

## Experimental Section

### Materials and Strains

The probiotic *Bacillus coagulans* (BC) was obtained from BeNa Chuang Lian Biotechnology Research Institute (Beijing, China). Sodium alginate powder, bile salts, and nitrate reductase (NR) test kit were provided by Solarbio (Beijing, China). Citric acid and calcium chloride anhydrous (CaCl_2_) were purchased from Kermel (Tianjin, China). Sodium tungstate dihydrate (Na_2_WO_4_·2H_2_O) and wheat germ agglutinin conjugated with FITC (FITC-WGA) were purchased from Sigma-Aldrich (StLouis, USA). The Live & Dead Bacterial Staining Kit was bought from Shanghai yuanye Bio-Technology Co., Ltd. Dextran sulfate sodium (DSS, molecular weight, 36–50 kDa) was obtained from MP Biomedicals (California, USA). IL-6, IL-10, IL-1β, TNF-α, and TGF-β enzyme-linked immunosorbent assay (ELISA) kits were obtained from Shanghai Enzyme-linked Biotechnology Co., Ltd.

### The Preparation of CTM Composites

CTM was prepared by crosslinking a mixture of 3 wt % sodium alginate and 0.6 wt % Na_2_WO_4_·2H_2_O in CaCl_2_ solution. First, 0.3 g of Na_2_WO_4_·2H_2_O was dissolved in 50 mL water, and citric acid was added to adjust the solution pH = 7. 1.5 g sodium alginate powder was slowly added to the solution. The above mixed solution was placed in a warm bath at 45°C stirred continuously for 2 h to produce a homogeneous sodium alginate sodium tungstate hydrosol, and the hydrosol was poured into a beaker and cooled to 25°C. Subsequently, the hydrogel was completely immersed in the 5% CaCl_2_ solution to trigger the crosslinking reaction.

### Characterizations of CTM Composites

The morphology of CTM was evaluated by scanning electron microscopy (SEM, Hitachi) and confocal laser scanning microscopy (CLSM, LeicaTCSSP, Germany). The species and distribution of CTM elements were analyzed by energy dispersive X-ray spectrometry (EDS). Fourier transform infrared (FT-IR) spectra of CTM were obtained using an infrared spectrophotometer (JASCO). The crystal structure of CTM was identified by Bruker AXS D2 X-ray diffraction (XRD). The chemical composition of CTM was examined by X-ray photoelectron spectroscopy (XPS). The rheological analysis of CTM was performed by a Discovery series hybrid rheometer-1 (TA Instruments, DHR-2, USA). The zeta potential of CTM was analyzed by dynamic light scattering (DLS, Malvern).

### Preparation of BC@CTM or CTM

To obtain an emulsion containing sodium tungstate alginate, 5 mL of sodium tungstate alginate solution was obtained using the same method as above with or without adding 2.5 mL of probiotic solution (∼10^8^ CFU mL^–1^). The mixture was stirred for an additional 30 min, and then 5 mL of the oil phase was added to the final concentration of 2 wt % Tween 80. The w/o emulsion was produced using a mechanical stirrer at 450 rpm. Another emulsion containing CaCl_2_, containing a 5% (w/o) CaCl_2_ solution, was prepared in the same manner as the first emulsion. Under stirring at 450 rpm, 10 mL of CaCl_2_ emulsion was added dropwise to 10 mL of sodium alginate tungstate probiotic emulsion to produce microcapsules after 15 min. Afterward, acetic acid solution (pH = 5.5) was added to dilute the emulsion, after which the emulsion containing microcapsules was stirred at 200 rpm for 15 min and left for 1 h.

### Encapsulation Efficiency of BC

The resulting hydrogel prepared in the previous step was sterilized (121°C for 15 min) prior to the encapsulation procedure. After cooling to room temperature, 2 mL of BC (1 × 10^8^ CFU mL^–1^) was mixed with 4 mL of the hydrogel solution and stirred for 30 min. Finally, the suspension was injected drop by drop into the CaCl_2_ crosslinking solution via sterile syringe. The CTM-encapsulated BC were collected, washed with distilled water, filtered, and sealed in sterile conical tubes for the following experiments. The encapsulation rate (EE) of BC was calculated as follows:
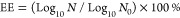
where *N* and *N*_0_ represent the live CFUs before and after encapsulation.

### The Swelling Ratio of Dried CTM

The swelling ratio of the CTM was measured under pH 1.2, 6.8, and 7.4, respectively at 37°C, respectively for 120 min. The CTM was removed from the solution at 120 min, and the surface fluid was cleared with filter paper before weighing. The swelling rate was determined according to the following equation:
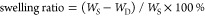
where *W*_S_ refers to the weight of swollen CTM, and *W*_D_ is the weight of dried CTM.

### To Observe the Stability of CTM in SGF and SIF

CTM (200 μL) in physiological saline was added into 800 μL artificial gastric fluid (SGF, pH = 1.2, containing 20 mg NaCl, 32 mg pepsin and 70 μL hydrochloric acid per 100 mL ddH_2_O). The mixed solution was incubated at 37 °C with a gentle stirring at 60 rpm. Then, centrifugation at 12000 *g* for 5 min was performed, and the supernatant was discarded. CTM (200 μL) in physiological saline was added into 800 μL artificial intestinal fluid (SIF, pH = 7.4) to the pellet and incubated for another hour at 37°C, 60 rpm. It was taken out for CLSM and SEM to observe the morphology and the autofluorescence of CTM.

### CTM with CP-Responsive Release Properties

The release of tungsten was assayed by using simulated gastric and simulated intestinal fluids. Two 25 mg of CTM were taken and soaked first in 5 mL of SGF for 6 h and then in SIF with or without CP for 24 h. At certain time intervals, 500 μL of the solution was removed and supplemented with an equal amount of intestinal fluid. Finally, the extracted solutions were centrifuged, and the supernatant was taken for the quantitative analysis of tungsten release by applying inductively coupled plasma mass spectrometry (ICP-MS, Thermo Fisher). To simulate the release of tungsten from CTM at high concentrations of CP in colitis, two portions of CTM were taken and soaked first in 5 mL of SGF for 6 h and then in SIF with or without 500 μg mL^–1^ of CP for 24 h. After centrifugation, the supernatant was quantified for tungsten release using ICP-MS.

### Release Concentration of Tungsten in the Colon

To measure the release concentration of tungsten in the colon, we administered equal amounts of CTM to healthy and colitis mice for 6 h. After this, we ground 50 mg of colon tissue by adding 1 mL of saline, centrifuged it at 4000 rpm for 10 min, and quantified the tungsten concentration in the supernatant using ICP-MS.

### *In Vivo* CP Assay

To perform the *in vivo* CP assay, we ground 50 mg of colonic tissue from both healthy and colitis mice with 1 mL of saline. After centrifuging of the mixture at 4000 rpm for 10 min, we measured the concentration of calprotectin in the supernatant using a mouse calprotectin (CALP) ELISA kit.

### Nitrate Reductase Activity Detects and Anaerobic Growth Detects

To induce nitrate reductase expression, overnight cultures of BC, BS, *E. coli* K-12, or *E. coli* DH5α were 1:100 diluted in fresh NB or LB broth containing 40 mM sodium nitrate. Then Na_2_WO_4_·2H_2_O (0.2 mg L^–1^) or CP (50 μg mL^–1^) or CTM (0.2 mg L^–1^) or CTM (0.2 mg L^–1^) + CP (50 μg mL^–1^) was added to the broth medium. The cultures were incubated aerobically at 37 °C for 3 h, and the relative nitrate reductase activity was determined using an NR test kit. The bacteria were incubated anaerobically overnight at 37°C according to the same method described above before performing colony counts.

### Characterization of Encapsulated Probiotics

The CTM morphology of the encapsulated probiotics was visualized with SEM. Rhodamine-labeled CTM encapsulated BS-GFP colocalization was detected by CLSM.

### Growth Curve of Encapsulated Probiotics

Probiotics were released by smashing BC@CTM (1 mL) with magnetic stirring in the presence of 9 mL sodium citrate solution (5.0%). Using free BC as a control, live probiotics were diluted with NB broth to OD600 = 0.2 and incubated with 200 rpm shaking in a 37°C incubator. The OD600 values of probiotics were recorded at 1 h intervals by a microplate spectrophotometer (BioTek, UK). The difference in bacterial growth was further checked between BC@CTM and BC. Dilution plate colony counts were performed after incubating 1 mL of OD600 = 0.2 for 12 h.

### Bacterial Viability Assay Using CCK-8

BC@CTM and BC were taken in NB broth medium and diluted to OD600 = 0.5. 190 μL of each probiotic and 10 μL of CCK-8 (MedChemExpress) were added to a 96-well plate and incubated in an incubator at 37°C. The OD450 values of probiotics were then recorded hourly using a microplate spectrophotometer.

### *In Vitro* Bacterial Competitive Colonization

Mixtures of BC, CTM, CTM + CP, and *E. coli* (1:1:1:1) were cocultured in growth medium of SIF at 37°C. Subsequently, the mixture solution was harvested after 24 h incubation and spread on the following selective media: nutrient agar known for selecting isolation of BC, and agar known for selective isolation of *E. coli*. Finally, the culture plates were incubated at 37°C overnight for counting BC and *E. coli*, respectively.

### Resistance Assay In Vitro

BS@CTM and BS (1 × 10^8^ CFU) were resuspended in 2 mL SGF and cultured in an incubator with 200 rpm at 37 °C for 2 h. BS@CTM and BS were resuspended in 2 mL bile salt and cultured in an incubator with 200 rpm at 37 °C for 6 h. 50 μL of each sample was taken at specific time points, washed with phosphate buffer and spread on NB agar medium. The bacteria were cultured overnight in the incubator at 37 °C, and the colonies were counted. Meanwhile, a Live & Dead Bacterial Staining Kit was used to stain BC and visualized by CLSM. In addition, the same amount of BC@CTM and BC (1 × 10^8^ CFU) were incubated serially in 1 mL of SGF and bile salts to simulate the harsh GI environment. The solution was centrifuged and resuspended in broth medium and gently shaken at 37°C. The OD600 value was examined every 1 h, and the growth curves were plotted.

### Survival Rate in Mice

Five 8-week-old female BALB/c mice per group were dosed orally with 200 μL of BC or BC@CTM containing1 × 10^8^ CFU. At 4, 48 or 96 h, mice were sacrificed. The stomach, small intestine, colon, and cecum were harvested from mice to assess BC adhesion and colonization in the GI tract. 50 μL of homogenized tissue was spread on NB agar medium. The culture was incubated at 37°C for 12 h, followed by colony counting. Tissues were infiltrated in universal tissue fixation fluid containing 4% paraformaldehyde (1 mL per sample) for at least 24 h and processed into longitudinal sections. Samples were embedding and subsequently Gram stained.

### Biosafety Assessment

Mice were sacrificed on day 5 after administration of 200 μL of BC@CTM (1 × 10^8^ CFU mL^–1^). Samples of blood, heart, liver, spleen, lung, kidney, and intestinal tissues were then collected. Unencapsulated BC, PBS, CAM, and BC@CAM were used as controls. The samples were immersed in tissue fixative and then stained for H&E.

### The Distribution of Probiotics in the GI Tract

Fluorescence imaging *in vivo* (ami HTX, Spectral Instrument Image, USA) was used to observe the GI distribution after oral delivery of probiotics. BS-mCherry was incubated in 2 mL NB broth containing kanamycin sulfate (50 μg mL^–1^, Solarbio) overnight and dilute bacteria in PBS to 10^8^ CFU mL^–1^. 42 mice were randomly grouped into BS-mCherry and BS-mCherry@CTM. Every mouse was fed with a bacterial suspension of 200 μL. Three mice from each group were sacrificed at 1 h, 2 h, 4 h, 8 h, 12 h, 24 h and 48 h and the intestines were collected for imaging.

### Long-Term Adhesion of Probiotics *in Vivo*

8-week-old BALB/c mice after 1 week of acclimatization feeding were randomly divided into three groups. Mice were fed with 200 μL of BS-mCherry @CTM, BS-mCherry (1 × 10^8^ CFU mL^–1^), and PBS, respectively. The above mice were sacrificed on days 3 and 6 after intragastric administration to obtain colonies. Each colon was cut into 1 cm areas and then sectioned at a depth of 10 μm after freezing. Tissue sections were observed with CLSM.

### 3D Images of Mucus Permeability

To conduct 3D mucus permeation observation, the colon from male rats was obtained cut into 4 cm lengths, and a section was sealed with medical sutures and then cultured with 3% DSS solution at 37 °C for 48 h to simulate mucus damage in DSS colonized mice. 100 μL of FITC-WGA (10 μg mL^–1^) was added to stain the mucus. After excision of the colonic tissue, 200 μL bacterial suspension containing BS-mCherry or BS-mCherry@CTM (10^8^ CFU mL^–1^) was slowly added to the mucus surface. Lastly, after leaving for 1 h at 37 °C, the distribution of bacteria in the mucosal layer was measured by CLSM.

### Animals

7–8 weeks old female BALB/c mice were provided by Beijing HFK Bioscience Co. All animals were experimented in accordance with the Animal Ethics Committee of Zhengzhou University approved by “Zhengzhou University Guide for the Care and Use of Laboratory Animals.”

### DSS-Induced Colitis Model in Mice

8-week-old female BALB/c mice were fed for 1 week with 3% DSS as previously described. Mice were then randomly divided into 6 groups and orally administered CTM, BC (1 × 10^8^ CFU day^–1^), BC@CAM, BC@CTM, or PBS for 5 days. During the 12-day experiment, changes in body weight were assessed daily. To confirm the success of colitis model establishment, changes in disease activity index (DAI) were recorded daily during the establishment of the colitis mouse model. On the last day mice were sacrificed to collect blood and the whole colon was stained for H&E and immunofluorescence staining. Additional feces were collected for intestinal flora analysis.

### Histopathology Analysis

The colon was fixed in tissue fixative and then embedded in paraffin for H&E staining according to standard procedures. Finally, the staining results were observed by fluorescence microscopy.

### *In Vivo* Immunofluorescence Imaging

Anti-ZO-1 antibody conjugated to AlexaFluor-488 at 5 μg mL^–1^ and anticludin-1 antibody conjugated to AlexaFluor-594 at 1 μg mL^–1^ were used to stain colon tissue sections. Nuclei were stained with Hoechst, and TUNEL assays were also performed to detect apoptosis in the colon. Images were obtained by CLSM.

### *In vivo* Enzyme-linked Immunosorbent Assay (ELISA) Analysis

To determine the levels of cytokines in colon tissue, colon segments were homogenized in PBS at 4 °C (1:10 w/v). Each sample was centrifuged at 10000*g* for 10 min at 4 °C. The levels of IL-6, IL-10, IL-1β, TGF-β, and TNF-α in colon tissue were determined by ELISA.

### Intestinal Permeability Assay In Vivo

Intestinal permeability assays were performed using 4 kDa FITC-dextran (FD4, Sigma) as previously described *in vivo*. After 4 h of food and water deprivation, mice were given 0.6 mg g^–1^ body weight of FITC-dextran orally. Mice were sacrificed to obtain serum samples, and the fluorescence intensity of FITC was measured.

### 16S Sequencing and Microbiome Analysis

Two or three feces were collected from each mouse after treatment in a colitis mouse model and analyzed by 16S rDNA sequencing on the NovaSeq platform. α-Diversity was used to characterize with Shannon and Simpson indices, and β-diversity was analyzed with PCoA.

### Statistical Analysis

All data were expressed as mean ± SD. Differences considered statistically significant were performed by Student’s *t* test of GraphPad Prism 8.02 (*P < 0.05, **P < 0.01, and ***P < 0.001).

## Supporting Information Available

Supplementary Figures 1–27. Representative SEM images of CAM; The zeta potential of CaWO4, CAM, and CTM; Strain dependent rheology measurement of the CTM hydrogel; The modulus of the CTM; The content of tungsten in CTM; The swelling rate of CTM for different Ph; Morphological changes of CTM under simulated gastrointestinal fluid; Representative images of CTM after SIF treatment; The stability of calcium tungstate after CTM treatment with SIF and calcium tungstate nanoparticles in solution; The stability of tungstate nanoparticles after CTM was treated with SIF + CP; CP concentrations in colonic tissue of normal and colitis mice; Release of tungsten in the CTM under CP (500 μg mL^–1^); Release of tungsten from colonic tissue of normal and colitis mice 6 h after oral administration of CTM; Bacterial counting of BC and BC@CTM after 12 h of incubation; Bacterial viability of BC and BC@CTM; Survival number of bacteria in the stomach at 4 h after oral administration; The total amount of BC reserved in the gut; Representative Gram staining images; Quantitative analysis of the distribution of BS-mCherry and BS-mCherry@CTM in the gastrointestinal tract; Distribution of BS-mCherry, BS-mCherry@CTM in other major organs; Daily weight change of each group for 5 days; H&E staining of the intestine, heart, liver, spleen, lung and kidney; H&E staining histology score evaluation of colon tissues; ROS level detected in the colon; Effects of BC@CTM, BC@CAM, and DSS on gut microbiota at genus levels using ternaryplot method; The LDA effect size analysis of BC@CTM and DSS; The LDA effect size analy of BC@CTM and BC@CAM (PDF)
