# Supplementary material for: Calcium Tungstate Microgel Enhances the Delivery and Colonization of Probiotics during Colitis via Intestinal Ecological Niche Occupancy
Source: ACS Cent Sci. 2023 Jun 21;9(7):1327–41. doi: 10.1021/acscentsci.3c00227 (PMC10375893; doi:10.1021/acscentsci.3c00227)
Supplement: Supplementary file 1 — oc3c00227_si_001.pdf [file oc3c00227_si_001.pdf]

Supporting Information for

Calcium tungstate microgel enhancing delivery and colonization of probiotic via intestinal ecological niche occupancy in colitis

Jiali Yang[a,b‡], *Mengyun Peng*[a,b‡], *Shaochong Tan*[a,b], *Shengchan Ge*[a,b], *Li Xie*[a,b], *Tonghai Zhou*[a,b], *Wei Liu*[a,b], *Kaixiang Zhang*[a,b,c], *Zhenzhong Zhang*[a,b,c *], *Junjie Liu*[a,b,c *], *Jinjin Shi*[a,b,c,d *]

[a] School of Pharmaceutical Sciences, Zhengzhou University, Zhengzhou 450001, P. R. China

[b] Key Laboratory of Targeting Therapy and Diagnosis for Critical Diseases, Zhengzhou 450001, P. R. China

[c] Collaborative Innovation Center of New Drug Research and Safety Evaluation, Zhengzhou

450001, P. R. China

[d] State Key Laboratory of Esophageal Cancer Prevention & Treatment, Zhengzhou, 450001, P. R. China

‡These authors contributed equally to this work.

*Corresponding author: Prof. Zhenzhong Zhang, Prof. Junjie Liu and Prof. Jinjin Shi

Email: zhangzhenzhong@zzu.edu.cn; liujunjie@zzu.edu.cn; shijinyxy@zzu.edu.cn

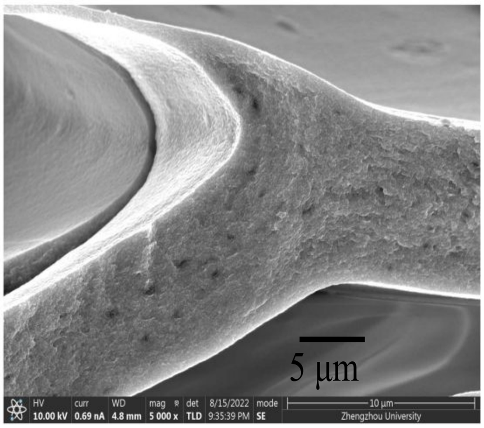

Figure S1. Representative SEM images of CAM.

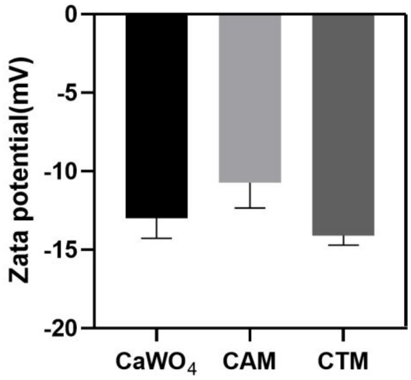

Figure S2. The zeta potential of $CaWO_4$, CAM, and CTM (n=3).

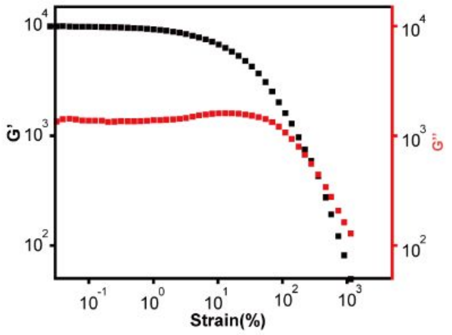

Figure S3. Strain dependent rheology measurement of the CTM hydrogel with the strain sweeping from 0.1% to 1,000% at an angular frequency of 10 rad/s.

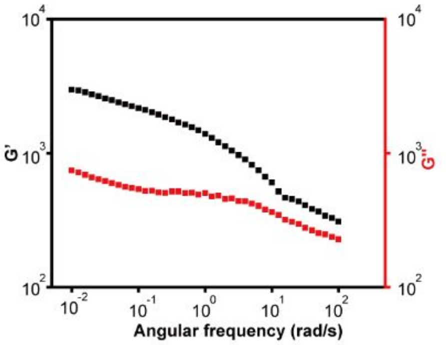

Figure S4. The modulus of the CTM changes as the frequency increases.

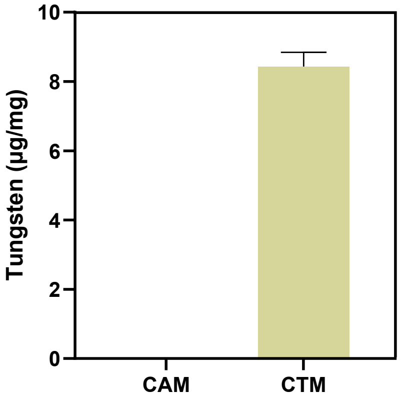

Figure S5. The content of tungsten in 1mg CTM using ICP-MS.

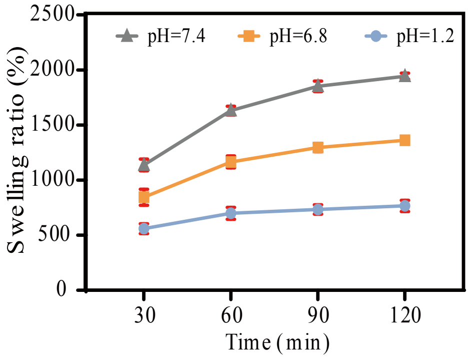

Figure S6. The swelling rate of CTM for different pH.

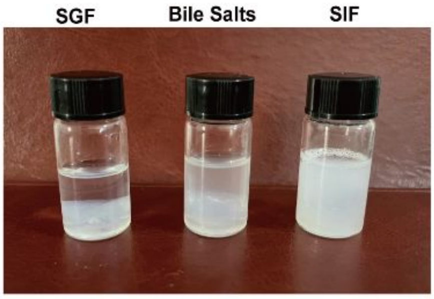

Figure S7. Morphological changes of CTM under simulated gastrointestinal fluid.

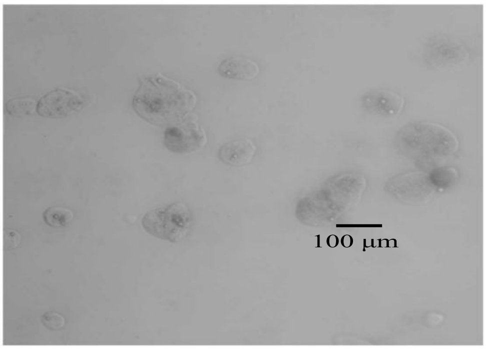

Figure S8. Representative images of CTM after SIF treatment.

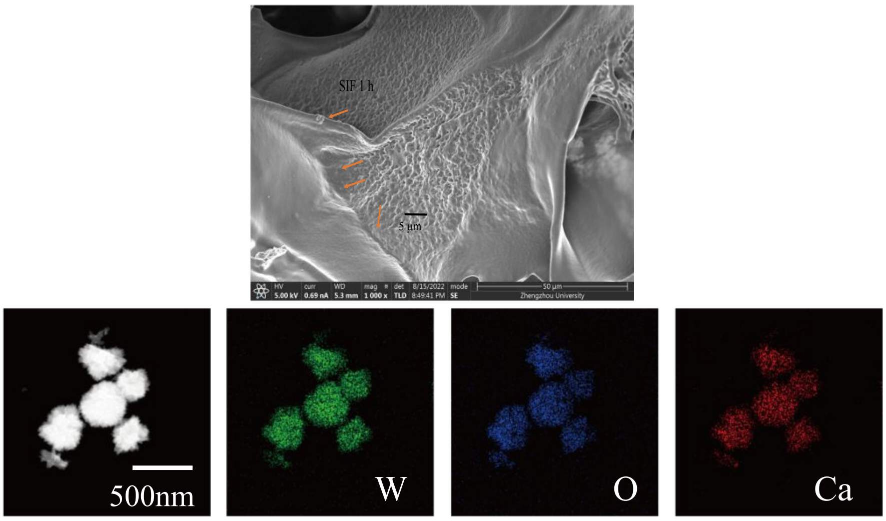

Figure S9. Stability of calcium tungstate after CTM treatment with SIF and calcium tungstate nanoparticles in solution (the arrow represents calcium tungstate nanoparticles).

SIF+CP 1 h

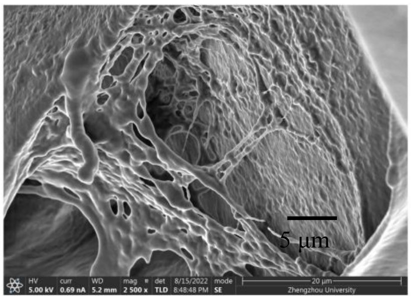

Figure S10. The stability of tungstate nanoparticles after CTM was treated with SIF + CP.

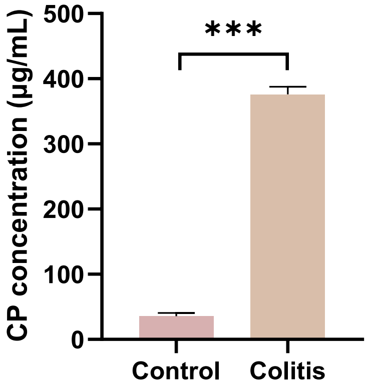

Figure S11. CP concentrations in colonic tissue of normal and colitis mice. Data are presented as the means ± SD (n = 3). **P < 0.05, **P < 0.01, ***P < 0.001* determined by Student's *t*-test.

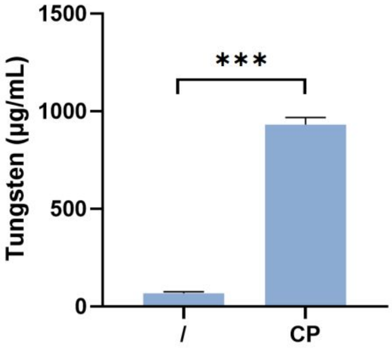

Figure S12. Release of tungsten in the CTM under CP (500 μg mL^{-1}). Data are presented as the means ± SD (n = 3). **P < 0.05, **P < 0.01, ***P < 0.001* determined by

Student's *t-test.*

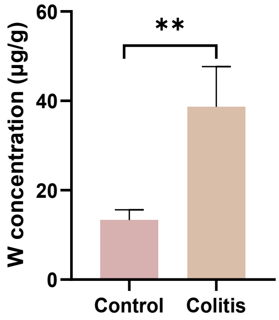

Figure S13. Release of tungsten from colonic tissue of normal and colitis mice 6 h after oral administration of CTM. Data are presented as the means ± SD (n = 3). **P < 0.05, **P < 0.01, ***P < 0.001* determined by Student's *t-test.*

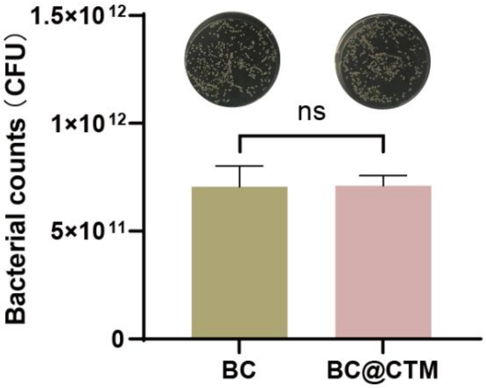

Figure S14. Bacterial counting of BC and BC@CTM after 12 h of incubation. Data are presented as the means ± SD (n = 3). **P < 0.05, **P < 0.01, ***P < 0.001* determined by Student's *t-test.*

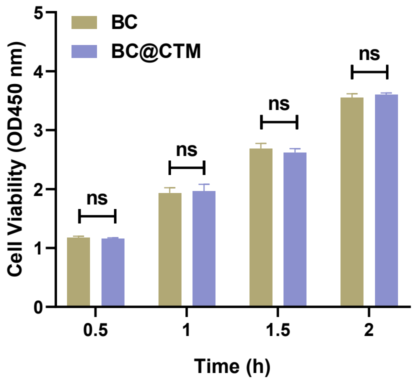

Figure S15. Bacterial viability of BC and BC@CTM was monitored by measuring OD450 at 1 h interval (n = 3). Data are presented as the means ± SD.

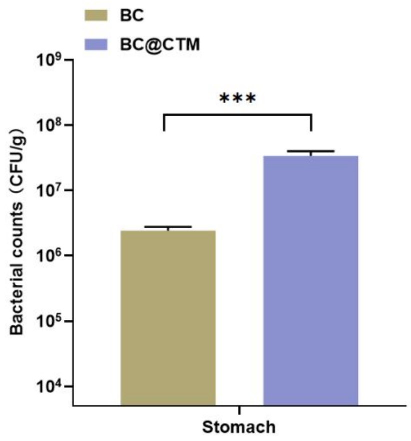

Figure S16. Survival number of bacteria in the stomach at 4 h after oral administration. Data are presented as the means ± SD (n = 3). **P < 0.05, **P < 0.01, ***P < 0.001* determined by Student's *t-test.*

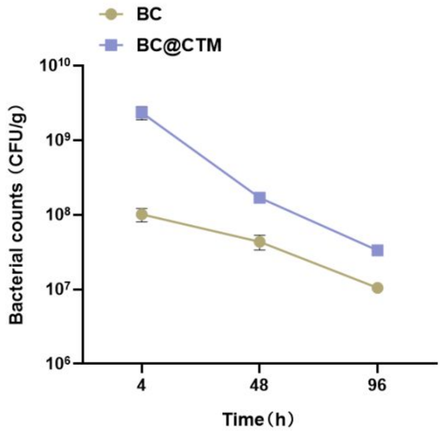

Figure S17. The total amount of BC reserved in the gut. Each mouse was orally administrated with 1 × 10^8 CFU of BC or BC@CTM and then executed at 4, 48, and 96 h. The contents extracted from the tissues were serially diluted with PBS, and 50 μL of each dilution was spread on the NB agar plates with kanamycin. Then the colonies were counted after incubation overnight at 37°C. Data are presented as the means ± SD (n = 3). *$*P < 0.05$, $**P < 0.01$, $***P < 0.001$* determined by Student's *t-test.*

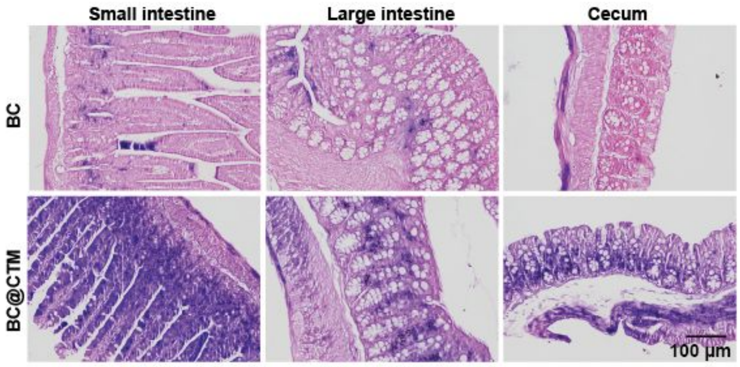

Figure S18. Representative Gram staining images. The colonization of BC and BC@CTM in the intestinal tract.

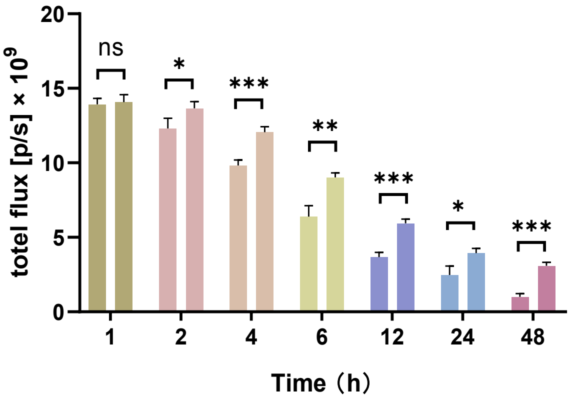

Figure S19. Quantitative analysis of the distribution of BS-mCherry and BS-mCherry@CTM in the gastrointestinal tract within 48 h after oral administration. Data are presented as the means ± SD (n = 3). $*P < 0.05$, $**P < 0.01$, $***P < 0.001$

determined by Student's *t-test.*

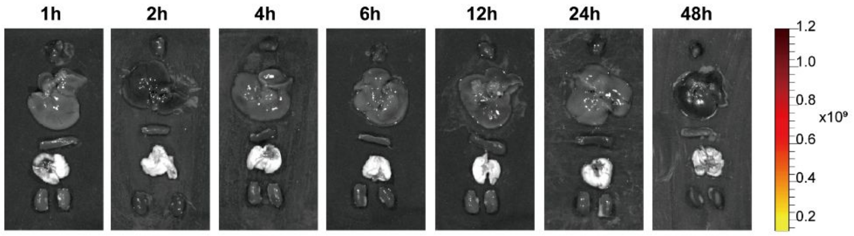

Figure S20. Distribution of BS-mCherry, BS-mCherry@CTM in other major organs (heart, liver, spleen, lung and kidney) within 48 h after oral administration by intestinal transit assay.

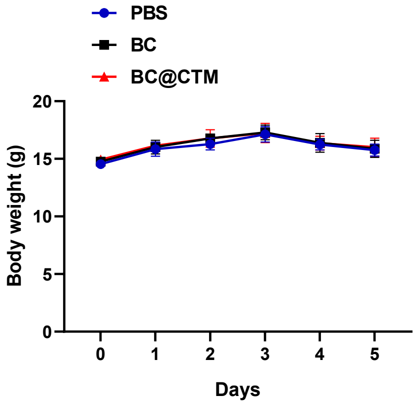

Figure S21. Daily weight change of each group for 5 days. Data are presented as the means ± SD (n = 3). $*P < 0.05$, $**P < 0.01$, $***P < 0.001$ determined by Student's *t-test.*

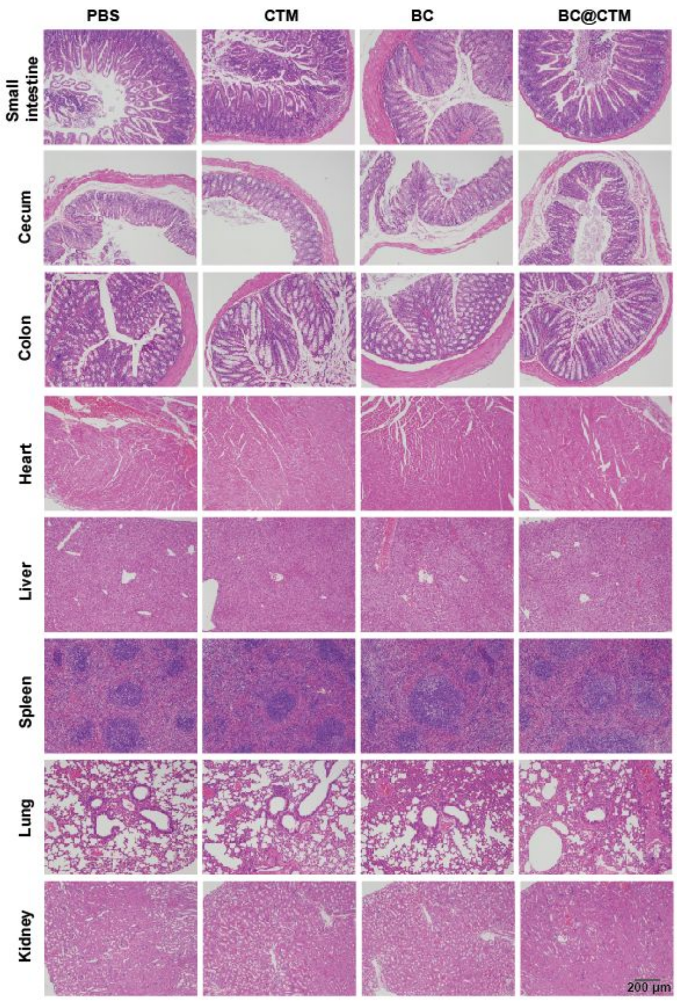

Figure S22. H&E staining of the intestine, heart, liver, spleen, lung and kidney. Tissues were obtained from mice at 5 day after administration of 10^8 CFU of BC, BC@CTM and CTM. PBS was used as a control.

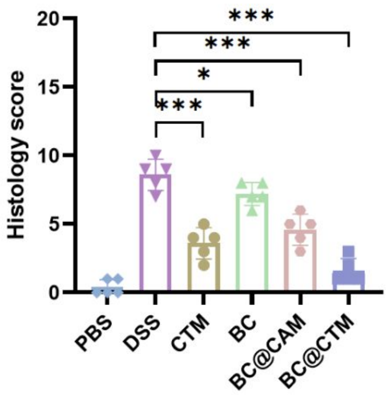

Figure S23. H&E staining histology score evaluation of colon tissues. Data are

presented as the means ± SD (n = 5). **P < 0.05, **P < 0.01, ***P < 0.001*

determined by Student's *t-test.*

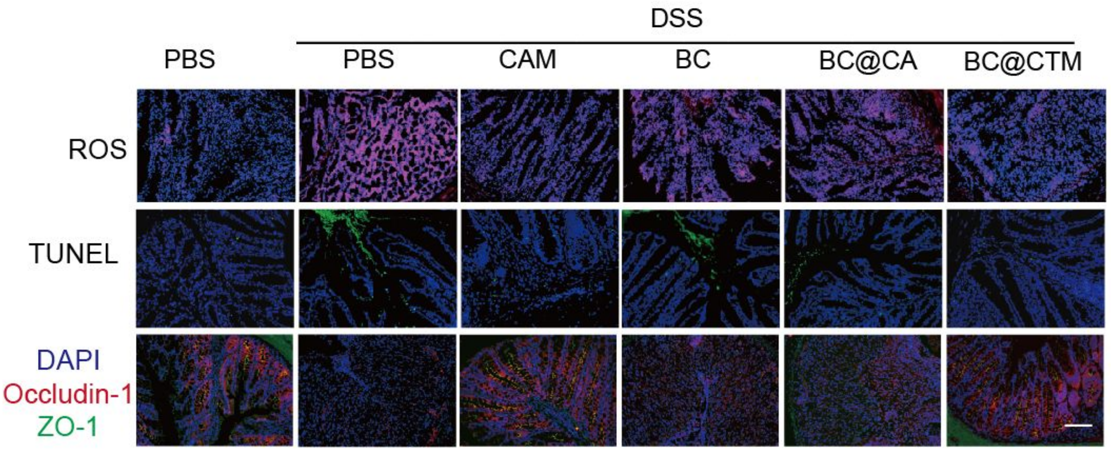

Figure S24. ROS level detected in the colon using DCFH-DA. Representative TUNEL staining images of the colon tissue. Representative immunofluorescence images of the expression of ZO-1 and occludin-1 in the colon. Scale bar: 100 μm.

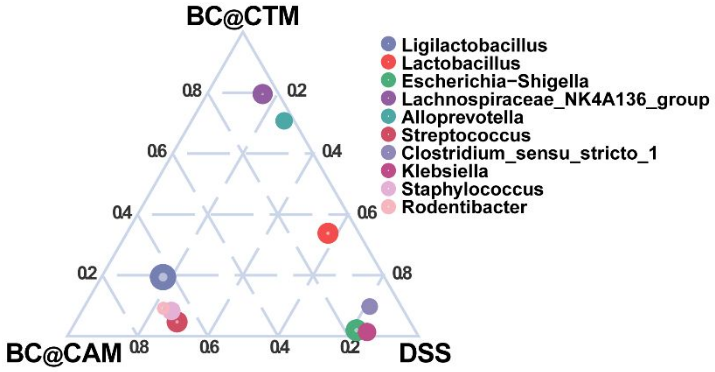

Figure S25. Effects of BC@CTM, BC@CAM, and DSS on gut microbiota at genus levels using ternaryplot method.

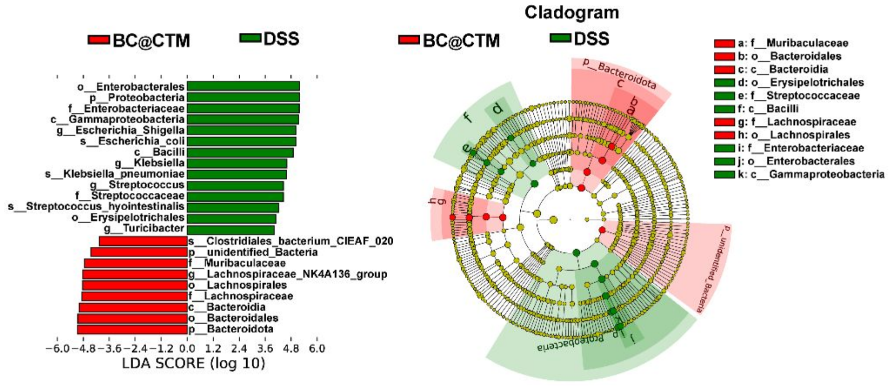

Figure S26. The LDA effect size analysis of BC@CTM and DSS.

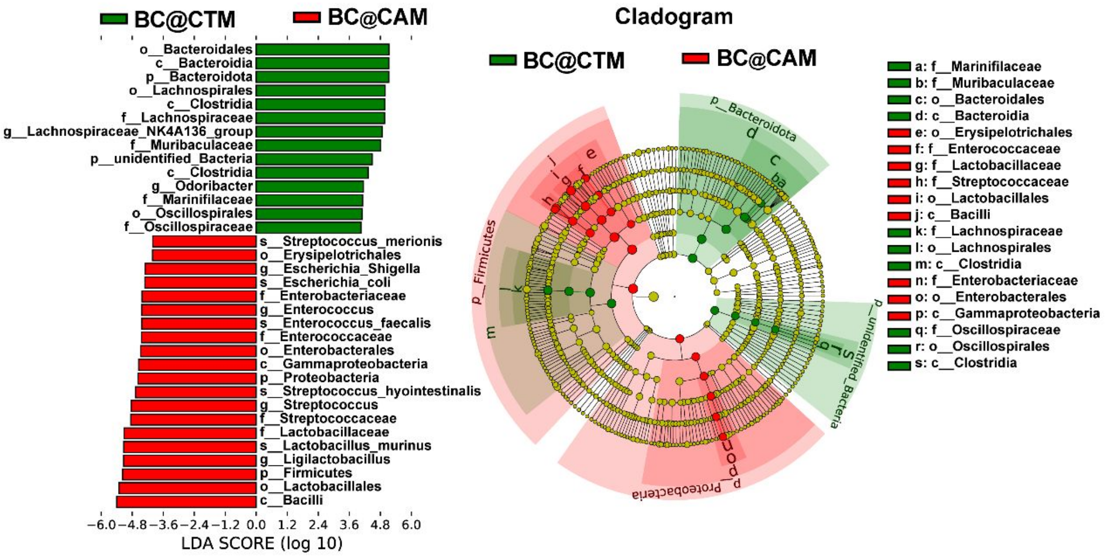

Figure S27. The LDA effect size analy of BC@CTM and BC@CAM.
